# Clinical Outcome of Hospitalized COVID-19 Patients with History of Atrial Fibrillation

**DOI:** 10.3390/medicina58030399

**Published:** 2022-03-07

**Authors:** Vincenzo Russo, Angelo Silverio, Fernando Scudiero, Antonello D’Andrea, Emilio Attena, Gisella Di Palma, Guido Parodi, Valentina Caso, Stefano Albani, Gennaro Galasso, Egidio Imbalzano, Paolo Golino, Marco Di Maio

**Affiliations:** 1 Cardiology Unit, Department of Translational Medical Sciences, University of Campania “Luigi Vanvitelli”—Monaldi Hospital, 80131 Naples, Italy; valepica8@yahoo.it (V.C.); paolo.golino@unicampania.it (P.G.); 2 Department of Medicine, Surgery and Dentistry, University of Salerno, 84084 Baronissi, Italy; angelosilverio1988@gmail.com (A.S.); ggalasso@unisa.it (G.G.); marcodimaio88@gmail.com (M.D.M.); 3 Cardiology Unit, Health Authority Bergamo East, 24121 Bargamo, Italy; fernandoscudiero@gmail.com; 4 Cardiology and Intensive Care Unit, Umberto I Hospital, 84014 Nocera Inferiore, Italy; antonellodandrea@libero.it; 5 Cardiology Unit, Cotugno Hospital, 80131 Naples, Italy; emilioattena@hotmail.it; 6 Medicine Unit, Santa Maria di Loreto Nuovo Hospital, 80142 Naples, Italy; gisella80@inwind.it; 7 Clinical and Interventional Cardiology, Sassari University Hospital, 07100 Sassary, Italy; parodiguido@gmail.com; 8 Cardiology Department, Aosta Valley Health Authority, 11100 Aosta, Italy; albani.aosta@gmail.com; 9 Department of Clinical and Experimental Medicine, University of Messina, 98122 Messina, Italy; egidio.imbalzano@unime.it

**Keywords:** novel coronavirus, SARS-CoV-2, COVID-19, atrial fibrillation, mortality, outcome

## Abstract

*Background and objectives*: Pre-existing atrial fibrillation (AF) is a frequent comorbidity in hospitalized patients with COVID-19; however, little is still known about its prognostic role in infected patients. The aim of our study was to evaluate whether the pre-existing AF as comorbidity would contribute to increase the risk for severe forms of COVID-19, worse prognosis, or even higher mortality. *Materials and Methods*: We retrospectively evaluated all consecutive COVID-19 patients admitted to the emergency department of nine Italian Hospitals from 1 March to 30 April 2020.The prevalence and the type of pre-existing AF have been collected. The correlation between the history and type of AF and the development of severe ARDS and in-hospital mortality has been evaluated. *Results*: In total, 467 patients (66.88 ± 14.55 years; 63% males) with COVID-19 were included in the present study. The history of AF was noticed in 122 cases (26.1%), of which 12 (2.6%) with paroxysmal, 57 (12.2%) with persistent and 53 (11.3%) with permanent AF. Among our study population, COVID-19 patients with AF history were older compared to those without AF history (71.25 ± 12.39 vs. 65.34 ± 14.95 years; *p* < 0.001); however, they did not show a statistically significant difference in cardiovascular comorbidities or treatments. Pre-existing AF resulted in being independently associated with an increased risk of developing severe ARDS during the hospitalization; in contrast, it did not increase the risk of in-hospital mortality. Among patients with AF history, no significant differences were detected in severe ARDS and in-hospital mortality between patients with permanent and non-permanent AF history. *Conclusions*: Pre-existing AF is a frequent among COVID-19 patients admitted to hospital, accounting up to 25% of cases. It is independently associated with an increased risk of severe ARDS in hospitalized COVID-19 patients; in contrast, it did not affect the risk of death. The type of pre-existing AF (permanent or non-permanent) did not impact the clinical outcome.

## 1. Introduction

Coronavirus disease 2019 (COVID-19) is the infectious disease caused by the severe acute respiratory syndrome coronavirus 2 (SARS-CoV-2), a highly pathogenic human coronavirus responsible for an epidemic of devasting proportion [1]. During the first pandemic wave, Italy was one of the countries with the highest number of confirmed cases [2,3]. The incidence of atrial fibrillation (AF) may complicate the clinical course of COVID-19 [4], and it seems to negatively impact on the prognosis of hospitalized patients [5,6,7]. The pre-existing AF could potentially impact on the clinical outcome of hospitalized patients with critical illness because it may increase the overall body inflammation and reduce the ability to compensate the hemodynamic alterations of acute illness. Actually, few data have been reported about the prognosis of hospitalized COVID-19 patients with AF history. The aim of our study was to evaluate whether the history of AF as comorbidity and the type pre-existing AF would contribute to increase the risk for severe forms of COVID-19, worse prognosis, or even higher mortality.

## 2. Materials and Methods

### 2.1. Study Design and Population

We performed an observational retrospective analysis of consecutive COVID-19 patients admitted to nine Italian Hospitals from 1 March to 30 April 2020. The clinical characteristics, pharmacological therapy and pre-existing-type AF (paroxysmal, persistent, or permanent) have been collected. We dichotomized the study population into two groups according to the pre-existence of AF as comorbidity (no-AF history vs. AF history group). Moreover, we additionally dichotomized patients with AF history into those with and without permanent AF (Non-permanent vs. permanent AF history groups). The diagnosis of AF history was made on the basis of at least 1 AF episode recorded at 12-lead electrocardiographic (ECG) or continuous ambulatory ECG monitoring. Patients with incomplete baseline (*n*: 67) or follow-up data (*n*: 37) were excluded.

### 2.2. Clinical Outcomes

The differences in terms of occurrence of severe ARDS in need of intensive care unit (ICU) and in-hospital mortality have been evaluated. ARDS was defined according to the Berlin definition [8]. The severe form of ARDS based on the degree of hypoxemia was diagnosed when the ration between *arterial oxygen tension* (PaO_2_) and the fraction of inspired *oxygen* (FIO_2_) was ≤100 mm Hg with positive-end expiratory *pressure* (PEEP) ≥ 5 cm H_2_O. The number of patients who experienced thromboembolic complications and pulmonary embolism was recorded. This study was conducted according to the Declaration of Helsinki and approved by the institutional ethics committees. The requirement for informed consent from individual patients was waived due to the observational retrospective design of the study.

### 2.3. Statistical Analysis

The Kolmogorov–Smirnov and the Shapiro–Wilk test were used to test the distribution of continuous data. Mean ± standard deviation (SD) and median with interquartile range (IQR) were used to express the normally and non-normally distributed variables, respectively. Categorical variables were expressed as numbers and percentages. Student t-test and Mann–Whitney U test were used to compare the continuous normally and non-distributed variables, respectively. Chi-squared test, or Fisher exact test, when appropriate, was used to compare the categorical variables. To assess the impact of AF history on outcomes in the overall population and of the permanent AF among patients with AF history, logistic regression models were performed, and the results were expressed with the risk ratios (RR) and their 95% confidence intervals (CI). All pre-procedural covariates potentially related to the outcome were included in the propensity score weighting model Two different propensity score weighting models, including age, sex, smoking habit, chronic obstructive pulmonary disease (COPD), hypertension, diabetes, coronary artery disease (CAD), heart failure, obesity, dyslipidemia, stroke, and chronic kidney disease (CKD), were used to account for potential bias between the study groups (AF history vs. no AF history and permanent AF history vs. non-permanent AF history). After weighting, standardized mean differences were calculated to assess the balance for all covariates included in the propensity score models; values higher than 0.10 were considered significant for differences among groups. A *p*-value < 0.05 was determined to be significant. Statistical tests were performed in R version 3.5.1 (R Foundation for Statistical Computing, Vienna, Austria).

## 3. Results

We included in the present study 467 consecutive COVID-19 patients (mean age 66.88 ± 14.55 years; 63% males) followed for a median time of 28 days (IQR: 12–45).

**Figure 1:**
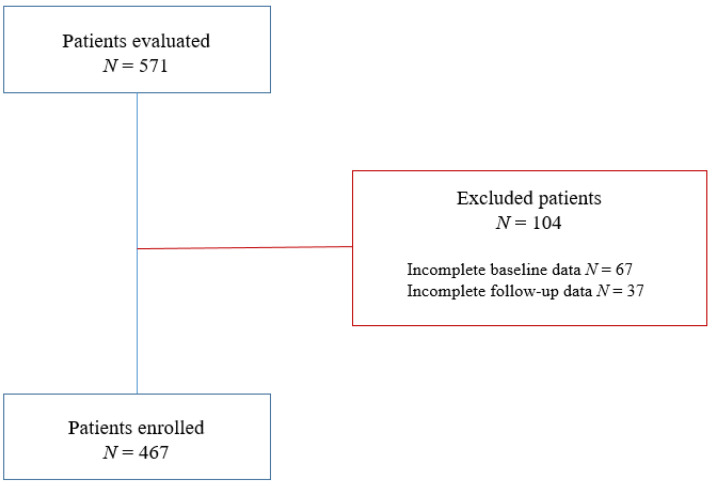
Inclusion graph of the study population.

The inclusion graph of the study population was shown in Figure 1.

The history of AF was noticed in 122 cases (26.1%), of which 12 (2.6%) had paroxysmal AF, 57 (12.2%) had persistent AF, and 53 (11.3%) had permanent AF. COVID-19 patients with pre-existing AF (AF history group) were older compared to those without pre-existing AF (No-AF history Groups) (71.25 ± 12.39 vs. 65.34 ± 14.95 years; *p* < 0.001) and were more likely to be taking anticoagulant therapy (40.9% vs. 11.0%; *p* < 0.001); however, they did not show statistical differences in cardiovascular comorbidities or treatments, except for the history of stroke (13.9% vs. 7.2%; *p* = 0.042). The clinical features of the study population are shown in Table 1. All enrolled patients were treated with the same therapeutic protocol based a triple combination including Lopinavir/Ritonavir (250/50 mg twice daily), Hydroxychloroquine (200 mg twice daily), and Azithromycin (500 mg once daily). All hospitalized patients showed fever and dyspnea.

**Figure 2:**
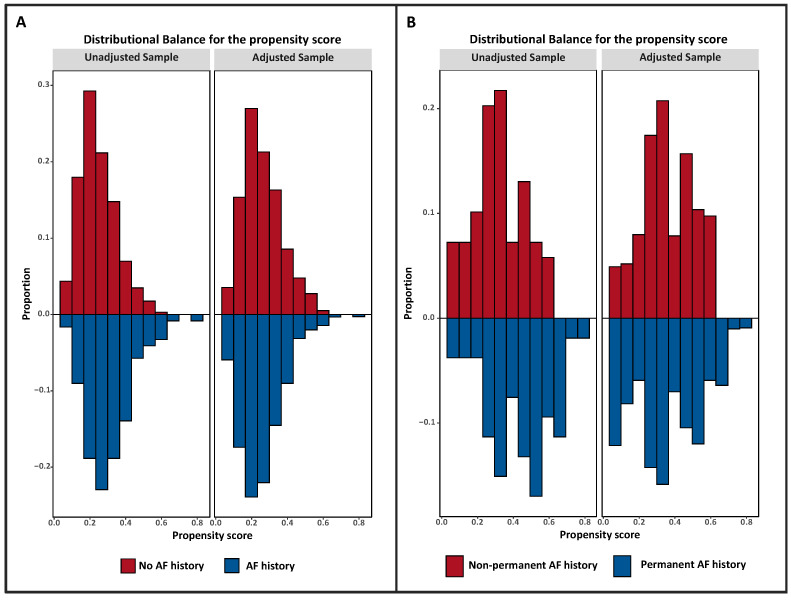
Distributional balance of the propensity score values before and after weighting between no AF history and AF history groups (**A**) and permanent vs. non-permanent AF history groups (**B**).

Among COVID-19 patients with AF history, those with permanent AF history were more likely older compared to those with non-permanent AF history (68.58 ± 11.17 vs. 74.74 ± 13.13 years; *p* = 0.006); no other significant differences were detected among the two groups (Table 2). The distributions of the propensity score values before and after weighting in pre-existing vs. non-preexisting AF and permanent vs. non-permanent AF history groups are plotted in Figure 2. A total of 7 out of 69 patients (10.1%) with non-permanent AF history and in sinus rhythm at admission switched to AF during hospitalization.

Among overall study population, 124 patients (26.5%) presented with severe ARDS at admission to the emergency department, 169 patients (36.2%) developed severe ARDS during hospitalization and 107 died (22.9%).

**Figure 3:**
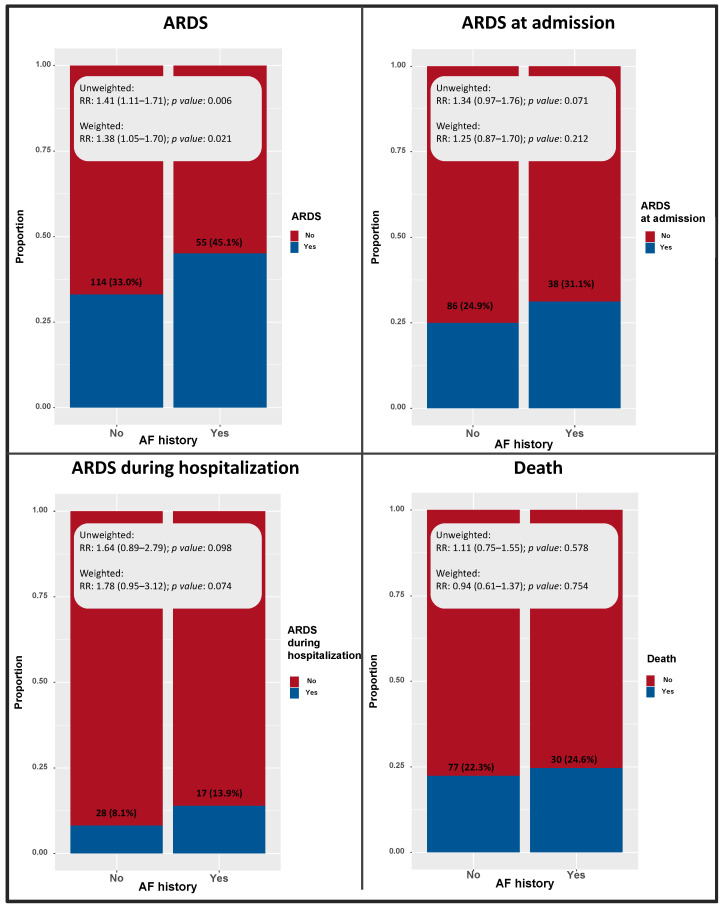
Prevalence and risk ratio of the outcome of interest among patients with and without AF history.

There was no statistically significant difference in severe ARDS at admission (31% vs. 24.9%, *p*: 0.189), severe ARDS during hospitalization (13.9% vs. 8.1%; *p* = 0.07), and overall mortality (24.6% vs. 22.3%, *p*: 0.604) between the AF history and No-AF history groups. At weighted analysis, the history of AF was significantly associated with increased risk of any ARDS (RR: 1.38; *p* = 0.021); a trend of association with ARDS during the hospitalization (RR: 1.78; *p* = 0.074) was shown; no remarkable associations with mortality among COVID-19 patients (RR: 0.94; *p* = 0.754) were found (Figure 3).

**Figure 4:**
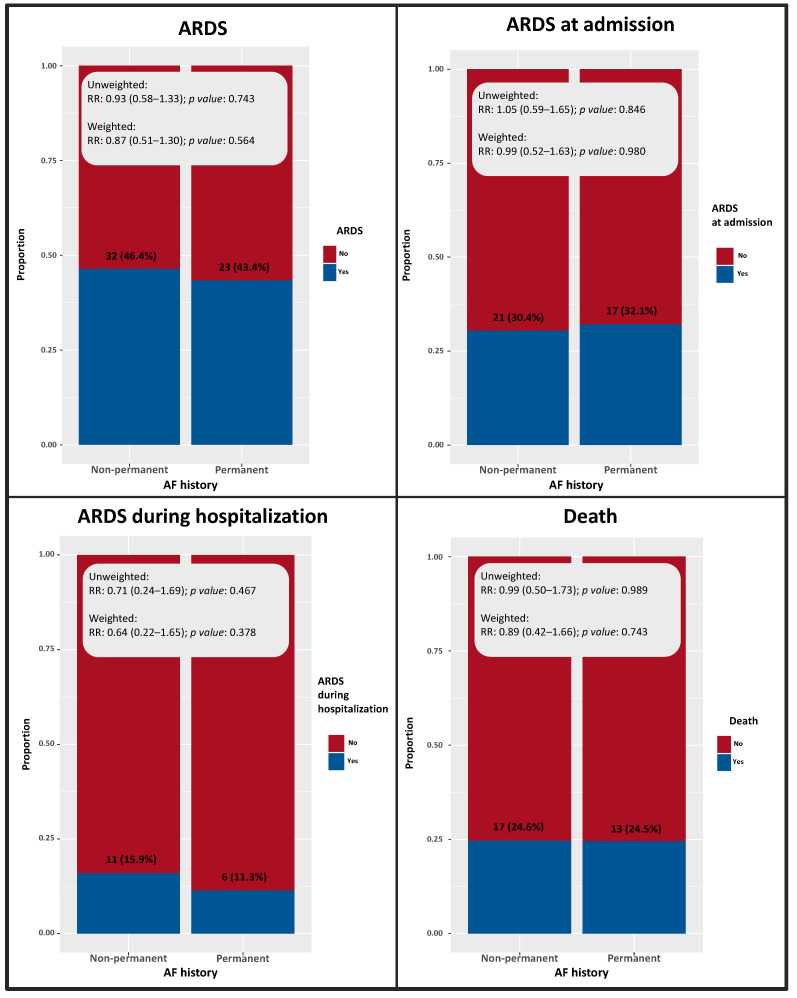
Prevalence and risk ratio of the outcome of interest among patients with non-permanent and permanent AF history.

Moreover, no significant differences in the incidence of outcome events were detected between patients with permanent and non-permanent AF history (Figure 4).

Among the overall study population, 88 patients were on oral anticoagulation at baseline. There was no statistically significant difference between patients with and without oral anticoagulant therapy in terms of ARDS at admission (26% vs. 28%, *p*: 0.834) and ARDS during the hospitalization (9% vs. 10%; *p* = 0.915). Among the No-AF history group, 51 patients (14.8%) experienced new-onset AF during hospitalization. There was no significant difference in terms of ARDS (at admission or during hospitalization) occurrence (40.7% vs. 31.5%, *p*: 0.173) and overall mortality (23.7% vs. 22.0%, *p*: 0.775) between No-AF history patients who experienced or not new-onset AF. Finally, we noticed 12 thrombotic complications (2.56%) during the hospitalization (4 stroke and 8 acute myocardial infarction) and 18 pulmonary embolisms (3.85%).

## 4. Discussion

The present study showed that 25% of hospitalized COVID-19 patients had personal history of atrial fibrillation, confirming that AF is a frequent comorbidity in this clinical setting. Moreover, pre-existing AF was independently associated with an increased risk of ARDS; in contrast, it did not affect the risk of in-hospital mortality. The type of pre-existing AF (permanent or non-permanent) did not impact the clinical outcome of hospitalized COVID-19 patients.

Despite the well-known association between cardiovascular diseases (CVD), including atrial fibrillation, and COVID-19 [9,10,11,12], little is known about the prognostic impact of pre-existing AF in hospitalized COVID-19 patients [13,14,15,16,17], and few data are available about the association between different forms of AF (permanent vs. non-permanent) and clinical outcomes [18].

Inciardi et al., among 99 patients hospitalized for COVID-19 in Northern Italy, reported an overall prevalence of 19%, which increases to 36% in those with cardiovascular diseases and 42% among patients who died during the hospitalization [13]; moreover, the history of AF was reported in 75% of geriatric patients with COVID-19 [14].

The data provided by the COVID-19 Task Force of the Italian National Institute of Health reported that AF was present in 24.5% of 355 non-surviving COVID-19 patients (mean age 79.5 years, 70% men) before the SARS-CoV-2 infection [15]. Moreover, the report of the New York State Department of Health showed that AF is the seventh among COVID-19 comorbidities [19].

Whether atrial fibrillation should be considered a cardiovascular risk factor associated with a worse prognosis in COVID-19 patients is still debated [20]; a low AF prevalence, about 3%, has been reported among COVID-19 patients not requiring hospitalization [21]. A recent metanalysis by Zuin et al. [18] including 15,562 COVID-19 patients across 12 different observational studies showed that pre-existing AF was associated with an increased risk of short-term mortality; however, the effects of the type of AF (permanent or non-permanent) were not investigated.

According to our results, the AF history seems to impact the clinical outcome of COVID-19 patients, increasing the risk of ARDS and suggesting that AF should be considered, among cardiovascular comorbidities, a red flag of worsening respiratory disease. The link between pre-existing AF and ARDS incidence in the clinical contest of COVID-19 might be related to the increased ACE2 expression in AF patients [22], which may promote the cell binding of SARS-CoV-2 and the inflammatory host response [23]. Based on this hypothesis, AF may be the arrhythmic marker of underlying inflammatory substrate favoring, and then amplified by, COVID-19 and leading to worse respiratory outcomes.

According to our results, the history of AF did not influence the risk of in-hospital mortality. These results diverge from the study by Gamst et al., who demonstrated a significant correlation between AF and mortality in a population-based cohort study including patients admitted in the intensive care unit [24]. Advanced age, male gender, ischemic heart disease, chronic kidney disease; some laboratory markers of oxygenation deficit, renal, and microvascular dysfunction; and coagulation activation have been significantly associated with the risk of in-hospital mortality [25,26]. Of note, the prevalence of these conditions was substantially lower in our population compared to what was reported by Gamst et al. [24] in the intensive care setting and may justify the discrepancy between the results of these studies. Recently, two metanalysis [27,28] suggested that COVID-19 patients with new-onset AF showed a significantly increased risk of all-cause mortality compared to those without AF; however, no data about the prognostic value of pre-existing AF have been provided. Our results suggest that the clinical outcome of hospitalized COVID-19 patients was not influenced by the type of pre-existing AF (permanent or non-permanent).

As previously showed [29,30], the preadmission antithrombotic therapy did not seem to show a protective effect in severe forms of COVID-19 with ARDS at presentation and rapidly evolving toward death. Among our study population, the incidence of thromboembolic events and pulmonary embolism was similar to those previously showed [4,17,31]. In the light of these results, we recommend following the international guidelines for vulnerable people living with chronic disease also for AF patients. Thereafter, careful clinical monitoring would be advisable in hospitalized COVID-19 patients with AF to early detect patients with a higher risk to develop ARDS during hospitalization.

## 5. Study Limitations

The present study has several limitations related to the observational retrospective design and the heterogeneity of COVID-19 clinical presentation. Although the study population was relatively small, we reported a high rate of adverse events to substantiate our analysis. To avoid the risk of overfitting due to an excess of variables examined compared to the number of events, we employed the propensity score weighting technique. Due to the absence of computed tomography imaging data, the COVID-19 severity was assessed on the basis of the presence of severe ARDS at admission. Further studies including a larger number of patients and different virus variants are needed.

## 6. Conclusions

Pre-existing atrial fibrillation is a frequent comorbidity in hospitalized COVID-19 patients, and it was independently associated to the ARDS but not to in-hospital mortality. The type of pre-existing AF (permanent vs. non-permanent) did not impact the clinical outcomes. Pre-existing AF should be considered a marker of increased vulnerability to worse respiratory disease during COVID-19. Careful monitoring and treatment are needed for this subgroup of patients.

## Figures and Tables

**Table 1:** Characteristics of the overall study population and differences between the two groups according to the presence or not of pre-existing AF.

	Overall	No AF History Group	AF History Group	*p*
*n*	467	345	122	
Male, *n* (%)	294 (63.0)	225 (65.2)	69 (56.6)	0.111
Age, mean (SD)	66.88 (14.55)	65.34 (14.95)	71.25 (12.39)	<0.001
Smoker, *n* (%)	79 (16.9)	62 (18.0)	17 (13.9)	0.378
Hypertension, *n* (%)	289 (61.9)	207 (60.0)	82 (67.2)	0.193
Diabetes, *n* (%)	123 (26.3)	86 (24.9)	37 (30.3)	0.296
Dyslipidemia, *n* (%)	119 (25.5)	92 (26.7)	27 (22.1)	0.386
Obesity, *n* (%)	35 (13.3)	28 (13.7)	7 (12.1)	0.924
Heart failure, *n* (%)	35 (7.5)	22 (6.4)	13 (10.7)	0.179
History of Stroke, *n* (%)	42 (9.0)	25 (7.2)	17 (13.9)	0.042
Chronic kidney disease, *n* (%)	62 (13.3)	41 (11.9)	21 (17.2)	0.182
Coronary artery disease, *n* (%)	71 (15.2)	58 (16.8)	13 (10.7)	0.139
Chronic Obstructive Pulmonary Disease, *n* (%)	90 (19.3)	67 (19.4)	23 (18.9)	0.997
Antiplatelet therapy, *n* (%)	141 (30.2)	100 (29.0)	41 (33.6)	0.400
Double antiplatelet therapy (%)	19 (4.1)	12 (3.5)	7 (5.7)	0.413
Oral anticoagulant therapy, *n* (%)	88 (18.8)	38 (11.0)	50 (40.9)	<0.001
Direct oral anticoagulant therapy, *n* (%)	50 (10.7)	15 (4.4)	35 (28.7)	<0.001
Vitamin K antagonist therapy, *n* (%)	32 (6.9)	18 (5.2)	14 (11.5)	0.032
Low molecular weight heparin, *n* (%)	135(28.9)	63 (18.2)	65 (53.3)	<0.001
Severe ARDS at admission, *n* (%)	124 (26.5%)	86 (24.9%)	38 (31%)	0.189
AF at admission, *n* (%)	62 (13.3)	1 (0.3)	61 (50.0)	<0.001
Permanent AF, *n* (%)	53 (11.3)	0 (0.0)	53 (43.4)	*-*
Non-permanent AF, *n* (%)	69 (14.8)	0 (0.0)	69 (56.6)	*-*
Persistent AF, *n* (%)	57 (12.2)	0 (0.0)	57 (46.7)	*-*
Paroxysmal AF, *n* (%)	12 (2.6)	0 (0.0)	12 (9.8)	*-*

**Table 2:** Characteristics and differences between the two groups according to history of non-permanent or permanent AF.

	Non-Permanent AF History Group	Permanent AF History Group	*p*
*n*	69	53	
Male, *n* (%)	39 (56.5)	30 (56.6)	0.999
Age, mean (SD)	68.58 (11.17)	74.74 (13.13)	0.006
Smoker, *n* (%)	8 (11.6)	9 (17.0)	0.557
Hypertension, *n* (%)	45 (65.2)	37 (69.8)	0.733
Diabetes, *n* (%)	23 (33.3)	14 (26.4)	0.532
Dyslipidemia, *n* (%)	14 (20.3)	13 (24.5)	0.735
Obesity, *n* (%)	3 (9.4)	4 (15.4)	0.769
Heart failure, *n* (%)	5 (7.2)	8 (15.1)	0.273
History of Stroke, *n* (%)	9 (13.0)	8 (15.1)	0.952
Chronic kidney disease, *n* (%)	12 (17.4)	9 (17.0)	0.999
Coronary artery disease, *n* (%)	6 (8.7)	7 (13.2)	0.614
Chronic Obstructive Pulmonary Disease, *n* (%)	10 (14.5)	13 (24.5)	0.241
Antiplatelet therapy, *n* (%)	24 (34.8)	17 (32.1)	0.904
Double antiplatelet therapy (%)	6 (8.7)	1 (1.9)	0.226
Oral Anticoagulant therapy, *n* (%)	28 (40.6)	21 (39.6)	0.999
Direct oral anticoagulant therapy, *n* (%)	19 (27.5)	16 (30.2)	0.905
Vitamin K antagonist therapy, *n* (%)	9 (13.0)	5 (9.4)	0.739
Low molecular weight heparin, *n* (%)	33 (47.8)	32 (60.3)	0.17
Severe ARDS at admission, *n* (%)	21 (30.4)	17 (32.1)	0.999

## Data Availability

The data presented in this study are available on request from the corresponding author.
